# Illness perceptions of gout patients and the use of allopurinol in primary care: baseline findings from a prospective cohort study

**DOI:** 10.1186/s12891-016-1252-z

**Published:** 2016-09-17

**Authors:** Ciaran P. Walsh, James A. Prior, Priyanka Chandratre, John Belcher, Christian D. Mallen, Edward Roddy

**Affiliations:** Research Institute for Primary Care & Health Sciences, Keele University, Staffordshire, ST5 5BG UK

**Keywords:** Allopurinol, Epidemiology, Gout, Illness perception, Primary care

## Abstract

**Background:**

Patients’ perceptions of their illness are dynamic and can directly influence aspects of management. Our aim was to examine the illness perceptions of gout patients in UK primary care and associations with allopurinol use.

**Methods:**

A health questionnaire was sent to 1805 people with gout aged ≥18 years identified by a gout diagnosis or prescriptions for allopurinol or colchicine in their primary care medical records in the preceding 2 years. The questionnaire included selected items from the revised illness perception questionnaire (IPQ-R). Associations between illness perceptions and use of allopurinol were calculated using multinomial logistic regression adjusted for age, gender, deprivation status, body mass index, alcohol consumption, comorbidities and gout characteristics.

**Results:**

One thousand one hundred eighty-four participants responded to the baseline questionnaire (65.6 %). Approximately half of responders perceived that they were able to control (51.2 %) or affect their gout through their own actions (44.8 %). Three quarters perceived treatments to be effective (76.4 %) and agreed that gout is a serious condition (76.4 %). Patients who agreed that they could control their gout (Relative Risk Ratio, 95 % confidence interval 1.66 (1.12 to 2.45)) and that treatments were effective (2.24 (1.32 to 3.81)) were more likely to currently be using allopurinol than not using allopurinol. However, this significance was attenuated after adjustment for self-reported gout characteristics (1.39 (0.89 to 2.17) & 1.78 (0.96 to 3.29) respectively).

**Conclusions:**

Patients who perceive that they can control their gout and that treatments are effective are more likely to be using allopurinol, this suggests that better information is needed for the patient from GPs and rheumatologist to reassure and support their use of ULT.

## Background

Gout is the most prevalent form of inflammatory arthritis, affecting 2.5 % of adults [1]. Gout is primarily attributed to hyperuricemia, the saturation of urate in and around the joints and connective tissues. Deposition of mono-sodium urate (MSU) crystals then leads to attacks of acute gout, tophi and progressive joint damage [2].

Long-term treatment of gout is achieved through urate-lowering therapies (ULT), such as allopurinol, which is the recommended drug for initial lowering of serum urate (sUA). Once target sUA levels have been reached, allopurinol use should be continued throughout life, to maintain sUA levels [3]. Despite these clear guidelines, primary care management of gout is typically sub-optimal in primary care [1].

When individuals are diagnosed with a chronic condition, such as gout, they will develop organised patterns of beliefs in response. Depending on how the individual perceives this new illness, these can directly influence aspects of management, such as drug adherence, but such illness perceptions are also dynamic and can alter [4]. Illness perceptions can be measured through rating and assessment of questionnaire statements related to the impact of illness on the individual, such as the ‘Revised Illness Perception Questionnaire’ (IPQ-R) [5]. Responses measure how the individual components of illness perception, for example *Identity* (Is illness central to how the individual identifies themselves?), *Cause* (How the disease arose?), interact to form the patient’s overall opinion regarding their chronic condition [6]. How one interprets the individual components forms an overall illness perception [7].

Illness perceptions are dynamic, with the potential to change depending on alterations in beliefs. Perceptions may change due to internal factors (e.g. beliefs about disease control) or external factors (e.g. effects of medication) and can potentially result in changes to disease outcomes [8]. De Gucht [9] found the relationship between irritable bowel symptom severity and separate quality of life dimensions to be fully mediated by illness perceptions. Dissatisfaction with treatment can manifest in follow-up consultations and lead to reduced adherence to treatment, leading to poorer disease outcomes [10]. Conversely an alteration in thoughts and emotions can also improve outcomes [4].

The relationship between illness perceptions and disease outcomes in gout has only previously been examined in a single study. Dalbeth et al. recruited 142 patients from primary and secondary care in New Zealand who completed the Brief Illness Perception Questionnaire (B-IPQ) and Health Assessment Questionnaire (HAQ II), at baseline and 1-year follow up. Participants who had poorer illness perceptions had an increased likelihood of disease progression and poorer adherence to ULT, suggesting that identification of such illness perceptions in clinical practice could be important to potentially identify those at risk of worse outcome [8]. However, the small sample size used in the Dalbeth et al. paper and the inclusion of secondary care patients, who would be likely to have more severe disease and hence different illness perceptions, are unlikely to be comparable to gout patients from UK primary care. 25 % of participants had tophi, which is more than typically seen in UK primary care.

There is presently little data on the illness perceptions of gout patients. Therefore, the aim of this cross-sectional analysis was to examine the illness perceptions of gout patients from UK primary care, with the specific objective to examine the associations between illness perceptions and the current use of allopurinol.

## Methods

### Study design and population

In 2012, as part of a longitudinal cohort study, a baseline questionnaire was mailed to 1805 gout patients, aged 18 years of age and older, registered with 20 general practices. Gout was defined by a history of previous gout consultation or a prescription of allopurinol or colchicine within the 2 years, identified in primary care medical records, prior to completing the baseline questionnaire [11]. This paper will describe cross-sectional analysis of baseline data from the cohort study. This study was approved by the North West - Liverpool East Research Ethics Committee (Ref no: 12/NW/0297).

### Survey measures

At baseline, characteristics recorded through the self-report questionnaire included age, gender, and body mass index (BMI), calculated from self-reported height and weight. Indices of Multiple Deprivation (IMD) were used to quantify deprivation status and categorise individuals into tertiles of; 20 % least affluent, 20 % most affluent and middle 60 %. We also recorded participants’ alcohol consumption.

Several gout specific characteristics were assessed, including; number of gout attacks in the previous 12 months, history of attacks of gout affecting more than one joint (oligo/polyarticular attacks), age at gout diagnosis, whether participants were currently experiencing a gout attack and whether they were currently taking allopurinol. Self-reported comorbidities were recorded, including; hypertension, transient ischaemic attack, anxiety, myocardial infarction, hyperlipidaemia, kidney stones and type II diabetes mellitus. The prevalence and severity of anxiety and depression were each recorded using a specific disease outcome, the generalised anxiety disorder (GAD-7) and patient health questionnaire (PHQ-9), respectively.

Questions were selected from the Revised Illness Perception Questionnaire (IPQ-R) [5], with participants answering several questions related to gout, comprising; i) *there is a lot I can do to control my gout?,* ii) *what I do will affect my gout?,* iii) *treatments are effective?,* iv) *gout is serious?,* in order to determine illness perceptions and patient understanding of disease.

### Statistical analysis

The characteristics of the study sample were initially summarised using descriptive statistics. The mean age (standard deviation (SD)) and gender were reported. BMI was categorised by those with a score <25 kg/m^2^ (healthy weight) or ≥25 kg/m^2^ (overweight). The frequency with which alcohol was consumed was categorised as; i) never, ii) occasionally, iii) 1–3 times per month, iv) 1–2 times a week, v) 3–4 times a week or vi) daily/almost daily. For gout-specific characteristics, number of gout attacks in the preceding 12 months was categorised into i) 0 gout attacks, ii) 1–3 gout attacks, iii) ≥4 gout attacks. History of oligo/polyarticular attacks and allopurinol use were categorised as yes or no. Possible IPQ-R responses were strongly agree, agree, uncertain, disagree or strongly disagree to the four statements. ‘Strongly agree’ and ‘Agree’ were combined into a single category (‘Agree’) and ‘Strongly disagree’ and ‘Disagree’ into a single category ‘Disagree’. Collapsing of categories was performed, boosting the number of responses in each of the three remaining categories and reducing extreme differences in response numbers between individual categories for each question.

Associations between the samples’ characteristics and the use of allopurinol were initially compared using independent *t*-test or chi-squared test (*χ*^2^). Multinomial logistic regression was used to identify associations between the use of allopurinol and IPQ-R responses, reported as relative risk ratios (RRR) with 95 % confidence intervals (95 % CI). After initial unadjusted analysis, RRR were adjusted for the sample characteristics of age, gender and deprivation status, BMI, alcohol consumption and comorbidities. The choice of these relating to their association with illness perceptions [8, 12] and gout [13, 14]. A subsequent adjustment was then made for gout characteristics, an important confounder likely to influence patients’ perceptions of their illness [8]. Gout characteristic included; the number of gout attacks in previous 12 months, history of oligo/polyarticular attacks, age at gout diagnosis and whether currently experiencing a gout attack.

Model diagnostics such as Nagelkerke’s Pseudo R^2^ and Pearson goodness-of- fit values are reported for the latter models in order to show that the models are a reasonable approximation of the data. Nagelkerke’s R-squared reflects the change in terms of log-likelihood from the intercept-only model to the current model. It does not convey the same information as the R-square for linear regression. All analyses were undertaken using SPSS v.21.

## Results

### Sample characteristics

Detailed characteristics of this sample have been reported elsewhere [15], but in brief, the 1184 responders to the baseline questionnaire (65.6 %) had a mean age of 65.6 years (SD = 12.5), the majority were male (83.6 %) and classified as overweight or obese (68.7 %). Nearly a quarter of participants drank alcohol on a daily basis (23.4 %) and the most prevalent comorbidities were hypertension (61.7 %), and hyperlipidaemia (42.9 %) (Table 1).

**Table 1:** General characteristics

		Using allopurinol	
Factor	Frequency (%)	No (%)	Yes (%)
Age Mean (SD)	65.6 (12.5)	65.1 (13.4)	65.9 (11.6)
Gender (Male %)	990 (83.6)	390 (79.6)	544 (86.4)
Alcohol Consumption			
Never	113 (9.7)	50 (10.4)	56 (9.0)
Occasionally	155 (13.3)	63 (13.1)	86 (13.8)
1–3 times per month	109 (9.3)	47 (9.8)	57 (9.0)
1–2 times per week	254 (21.8)	110 (22.9)	129 (20.6)
3–4 times per week	263 (22.5)	102 (21.3)	148 (23.7)
Daily	273 (23.4)	108 (22.5)	149 (23.8)
Body Mass Index			
Normal (<25 kg/m^2^)	371 (31.3)	168 (34.3)	178 (28.2)
Overweight/obese (≥25 kg/m^2^)	813 (68.7)	322 (65.7)	452 (71.8)
Neighbourhood Deprivation Status			
20 % least deprived	410 (34.6)	174 (35.5)	214 (34.0)
Mid-deprived	405 (34.2)	160 (32.7)	228 (36.2)
20 % most deprived	369 (31.2)	156 (31.8)	188 (29.8)
Depression (PHQ-9)	131 (11.1)	46 (11.0)	76 (13.4)
Anxiety (GAD-7)	109 (10.0)	42 (9.3)	59 (10.0)
Type II Diabetes Mellitus	205 (17.3)	60 (12.2)	138 (21.9)
Stroke	37 (3.1)	12 (2.5)	24 (3.8)
Hypertension	731 (61.7)	274 (55.9)	414 (65.7)
Transient Ischaemic Attack	62 (5.2)	24 (4.9)	34 (5.4)
Hyperlipidaemia	508 (42.9)	190 (38.8)	291 (46.2)
Kidney Failure	56 (4.7)	18 (3.7)	31 (4.9)
Myocardial Infarction	119 (10.1)	44 (9.0)	67 (10.6)
Kidney Stones	81 (6.8)	23 (4.7)	51 (8.1)
Angina	147 (12.4)	49 (10.0)	88 (14.0)

*SD* standard deviation, *PHQ-9* personalised health questionnaire-9, *GAD-7* generalised anxiety disorder 7 questionnaire

The average age at which gout had been diagnosed was 53.4 years (SD 15.9) and 725 (64.5 %) patients had experienced at least one gout attack in the previous 12 months. One hundred thirty-two patients (11.1 %) were experiencing an attack of gout at the time of completing the baseline questionnaire and 436 (36.8 %) had a history of oligo/polyarticular attacks. Just over half of baseline responders (56.3 %) were using allopurinol (Table 2). When stratified by allopurinol use, there was no difference in age, alcohol consumption, social deprivation or the prevalence of anxiety, depression, stroke, TIA, kidney stones or MI between users and non-users. However, allopurinol users were more likely than non-users to be overweight (*χ*^2^ = 4.70, df =1120 *p* = .030) and to have diabetes (*χ*^2^ (*1, N* = 1120) = 17.67, *p* = <0.001), hypertension (*χ*^2^ = 11.16, df =1120, *p* = .010), hyperlipidaemia (*χ*^2^ = 6.19, df = 1120, *p* = .013), kidney stones (*χ*^2^ = 5.17, df = 1120, *p* = .023) and angina (*χ*^2^ = 4.04, df = 1120, *p* = .044).

**Table 2:** Gout characteristics

		Using allopurinol	
Characteristics	Frequency (%)	No (%)	Yes (%)
Age at Gout Diagnosis (Mean, SD)	53.4 (15.9)	57.3 (15.5)	50.6 (15.5)
Number of attacks in previous 12 months			
0	398 (35.4)	96 (19.8)	298 (48.2)
1–3	521 (46.4)	253 (52.2)	163 (26.4)
≥4	204 (18.2)	136 (28.0)	157 (25.4)
Gout Attack at Present	132 (11.1)	54 (11.1)	69 (11.0)
History of oligo/Polyarticular gout	436 (36.8)	141 (29.0)	283 (45.3)
Currently Taking Allopurinol			
Yes	630 (56.3)	-	-
No	490 (43.7)	-	-

With regard to illness perceptions, approximately half of all participants agreed that there is either a lot they can do to control their gout (51.2 %) or that what they do will affect their gout (44.8 %). Three-quarters of gout patients perceived treatments to be effective (76.4 %) and agreed that gout is a serious condition (76.4 %).

Participants using allopurinol were diagnosed with gout at a younger mean age (50.6 years, SD 15.5) compared to non-users (57.3 years, SD 15.5), *t* (1076) = −7.11, *p* < 0.001 (Table 2). Allopurinol users were significantly less likely to have experienced one or more attacks of gout in the preceding 12 months (51.8 % versus 80.2 %) (*χ*^2^ = 95.63, df = 1103, *p* <0.001), but more likely to have a history of oligo/polyarticular attacks (45.3 % versus 29.0 %) than non-users (*χ*^2^ = 30.93, df =1103, *p* <0.001).

### Associations between illness perceptions and use of allopurinol

Unadjusted analysis showed that gout patients were more likely to agree (1.64 (1.14 to 2.34)) than disagree, with the statement that there is ‘*a lot I can do to control my gout’* when using allopurinol. This association was retained after initial adjustments (1.66 (1.12 to 2.45), but was attenuated after adjustments for gout characteristics (1.39 (0.89 to 2.17) (Table 3).

**Table 3:** Associations between Illness perceptions and allopurinol use

	Using allopurinol			Adjusted relative risk ratio (RRR) (95 % CI)	
	Yes (%)	No (%)	Unadjusted RRR (95 % CI)	Age, gender, deprivation, BMI, alcohol & co-morbidities	Age, gender, deprivation, BMI, alcohol, co-morbidities & gout characteristics
A lot I can do to control my gout					
Disagree (*N* = 154)	78 (12.7)	76 (16.2)	REF	REF	REF
Uncertain (*N* = 375)	190 (30.8)	185 (39.5)	1.00 (0.69 to 1.46)	1.01 (0.67 to 1.51)	1.10 (0.70 to 1.73)
Agree (*N* = 555)	348 (56.5)	207 (44.3)	**1.64 (1.14 to 2.34)**	**1.66 (1.12 to 2.45)**	1.39 (0.89 to 2.17)
Nagelkerke r^2^, Pearson GOF				**0.094,** ***p*** **= 0.053**	0.18, *p* = 0.43
What I do will affect my gout					
Disagree (*N* = 104)	64 (10.5)	40 (8.5)	REF	REF	REF
Uncertain (*N* = 487)	264 (43.3)	233 (49.4)	0.74 (0.48 to 1.14)	0.66 (0.41 to 1.04)	0.74 (0.44 to 1.23)
Agree (*N* = 480)	281 (46.2)	199 (42.1)	0.88 (0.57 to 1.36)	0.82 (0.51 to 1.31)	0.69 (0.41 to 1.16)
Nagelkerke r^2^, Pearson GOF				0.07, *p* = 0.65	0.12, *p* = 0.91
Treatments are effective					
Disagree (*N* = 75)	32 (5.2)	43 (9.3)	REF	REF	REF
Uncertain (*N* = 180)	73 (11.8)	107 (23.1)	0.92 (0.53 to 1.58)	0.98 (0.54 to 1.79)	1.06 (0.54 to 2.06)
Agree (*N* = 827)	513 (83.0)	314 (67.6)	**2.20 (1.36 to 3.54)**	**2.24 (1.32 to 3.81)**	1.78 (0.96 to 3.29)
Nagelkerke r^2^, Pearson GOF				**0.12,** ***p*** **= 0.11**	0.29, *p* = 0.54
Gout is serious					
Disagree (*N* = 63)	33 (5.3)	30 (6.4)	REF	REF	REF
Uncertain (*N* = 195)	98 (15.8)	97 (20.6)	0.92 (0.52 to 1.62)	0.79 (0.42 to 1.50)	0.91 (0.43 to 1.89)
Agree (*N* = 834)	490 (78.9)	344 (73.0)	1.29 (0.78 to 2.16)	1.15 (0.65 to 2.05)	1.15 (0.59 to 2.26)
Nagelkerke r^2^, Pearson GOF				0.09, *p* = 0.39	0.12, *p* = 0.96

Bold: *p* = <0.05. *GOF* goodness-of-fit

Gout patients were twice as likely to agree than disagree (unadjusted 2.20 (1.36 to 3.54), adjusted 2.24 (1.32 to 3.81)) that *‘treatments are effective’* if they were using allopurinol. However, this association was also attenuated when gout characteristics were adjusted for (1.78 (0.96 to 3.29). Both unadjusted and adjusted analysis showed no significant difference between the patient perception of ‘*What I do will affect my gout’* or that “*Gout is serious*” and their current use of allopurinol.

The Pearson goodness of fit *p*-values provided no real cause for concern, except the borderline result for the first adjusted model for ‘*a lot I can do to control my gout’*. Nagelkerke’s R-squared are quite low suggesting other variables might need to be recorded.

## Discussion

Our study examined the illness perceptions of gout patients from UK primary care and the associations between these perceptions and the current use of allopurinol. The majority of gout patients perceive that gout is a serious condition and that treatments are effective, but only half of patients believe that their actions have any influence over their own condition. Gout patients who feel more in control of their own condition and perceive treatments to be effective are more likely to be current users of allopurinol than non-users. Despite these associations being attenuated by gout characteristics, such links between illness perceptions and use of allopurinol remain worthy of reporting as this is likely an over-adjustment, due to the close associations between illness perceptions, use of allopurinol and severity of gout.

Our overall findings that patients perceived gout to be a chronic condition that can be managed with treatment, but for which personal actions have little influence are in-line with those of Dalbeth et al. [8]. Furthermore, Dalbeth et al. also reported issues between the perception of control and ULT, as patients who had concerns over ULT had less ability to control their disease and recorded higher symptomatic scores in the B-IPQ. This may be reflected in our findings that those who perceived less control of their gout were less likely to be using allopurinol.

Two key factors which impair the effectiveness of allopurinol are poor adherence and sub-optimal dosing to sufficiently lower urate levels [1]. Informing and empowering patients in the control of their own disease may improve subsequent managements, such as use of allopurinol [16]. It is clear that patients need to be better informed of the long-term benefits of allopurinol, such as preventing irreversible joint damage and complications associated with comorbidities [17].

The strengths of this study are that this uses a large sample of gout patients from UK primary care, adding to the limited data of illness perceptions in gout patients. This also builds on research examining the current role of allopurinol in UK primary care.

Limitations of this study include the use of only four IPQ-R questions from the responder questionnaire; however, these questions were selected on the basis that they most appropriately represented patient perceptions of gout and ULT, based on our previous qualitative research with gout patients [18]. One of these IPQ-R questions refers to treatment, but does not mention allopurinol or ULT specifically. In determining the perceptions of treatments in allopurinol users, we have assumed that patients taking allopurinol answered the treatment question with this in mind, rather than other treatments. As we specifically asked patients whether they were “currently taking a tablet called allopurinol”, then it is likely that this is their principal treatment (as guidelines recommend) and that they were at least adhering at the point of the baseline questionnaire. The vast majority of ULT users in primary care in the UK are prescribed allopurinol, with other ULTs (febuxostat and uricosurics) comprising less than 2 % of prescriptions for ULT in 2015 [19]. Whilst we believe that any perceptions about ULT will relate to allopurinol, we did not quantify use of, or ask about perceptions of other drugs such as colchicine or NSAIDs. We are also unable to determine whether the reasons behind non-allopurinol use are related to patient (e.g. poor adherence) or practitioner reasons (e.g. lack of provision by the general practitioner (GP)). Finally, our findings may be an effect of confounding by indication. It is likely that those prescribed allopurinol have the most severe gout, supported by our finding that allopurinol users more frequently reported experiencing oligo/polyarticular attacks in the past than non-users. Therefore, allopurinol may be acting as a proxy for increased disease severity.

Improved gout characteristics, be this due to allopurinol use or not, are the desired goals. If patients perceive that they are able to control and treat their disease, this may encourage improved adherence and continued optimal management. In light of these findings and that only half (53.2 %) of this gout sample were using allopurinol, the key clinical implication of our findings is a need to ensure that clinicians are appropriately trained to discuss the use of ULT with patients, and that patients are given high-quality information to aid their understanding of the benefits of ULT and importance of adherence to treatment.

## Conclusions

In conclusion, gout patients in UK primary care understand the seriousness of their condition and that treatments are effective, but perceive little personal control of their condition. Gout patients who viewed gout treatments as effective and did perceive a sense of control were more likely to be currently using allopurinol. Therefore, better information is needed for the patient, from GPs and rheumatologist, to encourage a sense of control over one’s condition with the aim improving health outcomes.

## Abbreviations

B-IPQ: Brief illness perception questionnaire
BMI: Body mass index
CLAHRC: Collaborations for leadership in applied health research and care
GAD: Generalised anxiety disorder
GP: General practitioner
HAQ II: Health assessment questionnaire
IMD: Indices of multiple deprivation
IPQ-R: Revised illness perception questionnaire
MSU: Monosodium urate
MTPJ: Metatarsophalangeal joint
NIHR: National Institute for Health Research
PHQ: Patient health questionnaire
R-IPQ: Revised illness perception questionnaire
RRR: Relative risk ratios
SD: Standard deviation
sUA: Serum urate
ULT: Urate-lowering therapies
